# The occupational health of Santa Claus

**DOI:** 10.1186/s12995-015-0086-1

**Published:** 2015-12-10

**Authors:** Sebastian Straube, Xiangning Fan

**Affiliations:** Division of Preventive Medicine, Department of Medicine, Faculty of Medicine and Dentistry, University of Alberta, 5-30 University Terrace, 8303-112 Street, Edmonton, AB T6G 2T4 Canada

**Keywords:** Santa Claus, Christmas, Occupational health

## Abstract

Previous publications in the field of Santa studies have not focused on health and safety issues arising from Santa’s workplace activities. However, it should be acknowledged that unique occupational hazards exist for Santa Claus. Major occupational health issues affecting Santa are discussed, along with suggestions for future research directions.

## Background

The health of Santa Claus has been discussed in the medical literature before [1] and it was concluded that “Santa studies is a developing field in public health, and currently there is a disappointing lack of rigorous research…” In particular, there is a lack of research on the occupational health of Santa Claus. Close examination of the working conditions of Santa Claus highlights a number of pertinent occupational health issues; we have attempted to outline the major points below.

## Main text

### Equipment operation

Sleigh safety is a point of concern, as, in the course of work, Santa encounters a number of transportation-related hazards that are not present for other workers in regulated transportation industries. Firstly, the analysis by Grills and Halyday [1] touches on safe (sleigh) driving, and in this context questions the tradition of leaving Santa a cup of brandy. Though there is uncertainty about whether Santa or the reindeer ought to be considered as the operator(s) proper of the sleigh, the consumption of alcohol during work would typically be discouraged, even if Santa were a sleigh passenger only. Additionally, as far as we are aware, there is a general lack of seatbelts and airbags on sleighs available to Santa, which are not manufactured to the engineering standards of the twenty-first century. Finally, Santa’s sleigh holds dual classification as a ground- and air-based transport vehicle, and, in the course of flight, Santa and the reindeer would be expected to contend with increasingly congested airspace above major cities and airports. Of note, Santa does not routinely travel with a copilot or radio operator, and it is unclear whether radio capability for monitoring standard aeronautical communication frequencies is standard-issue equipment on Santa’s sleigh, whether sleigh-mounted radar-based collision systems are available for Santa’s use, nor whether Rudolph the Red-Nosed Reindeer’s highly vascularized olfactory organ [2] functions adequately as a beacon light for other aircraft.

A recent study confirms that sleigh driving carries a high risk of injury [3]. We recall one disturbing report of Santa’s sleigh crashing on a remote tropical island, albeit during wartime (documented in the “I Saw Grampa Cussing Santa Claus” portion of the “Simpsons Christmas Stories” episode of “The Simpsons” [4]). In addition to the possibility of traumatic injuries to Santa or his reindeer which may be sustained during a sleigh crash, there are additional considerations of access to medical care and supplies in resource-poor settings like the one described, which the astute occupational health practitioner must be aware of and plan for in advance. In such an instance as an emergency crash-landing on a remote tropical island, it may be necessary to make use of locally available resources for medical care, for example coconut water for rehydration, which has even been used intravenously to good effect [5]. Of note, any physicians available to provide emergency medical care to Santa would likely need to be prepared to render aid without access to his entire medical record, which resides in paper form at the North Pole and has not been entered into any electronic health records as far as we are aware.

### Work scheduling, cardiovascular health and travel related issues

A further occupational health concern is that Santa’s work is rather unevenly distributed throughout the year. Little is known about what Santa does for most of the year, but he likely has to be considered “unemployed” for the largest portion. For the Christmas period, where his workload would demand that he exceed a typical eight-hour work day, Santa ought to be considered a shift worker who works a significant amount of seasonal overtime.

Santa, at least in popular depiction, displays two key cardiovascular risk factors: increased body-mass index (likely obesity), and a smoking habit at times. As these risk factors have been linked with unemployment and shift work [6–8], this is an example of adverse work conditions contributing to increased cardiovascular risk. Dietary patterns and metabolic responses to food have previously been established to be disrupted by shift work, and increased snacking has been seen in night shift workers, who are furthermore hampered in their efforts to maintain a healthy exercise balance [9]. It should be noted that Santa’s working hours during wintertime would be expected to fall within the hours of darkness at the North Pole. We note that Santa’s consumption of milk and cookies may exceed recommendations in most national nutritional guidelines (e.g., the Canada Food Guide [10]), and that it is unclear what he does for physical activity. Additionally, the nature of Santa’s work demands that a large volume of tasks be done in a limited period of time, likely leading to work stress, which has in itself been linked to increased cardiovascular risk [11].

It should be noted that, in addition to working long hours, Santa Claus traverses multiple time zones in the course of delivering presents to children in all parts of the world. We would expect Santa to suffer from significant jet lag due to the amount of travel required to meet his December 25^th^ deadline each year. Indeed, taking into account the transition between time zones, Santa is estimated to work some 36 h at a time consecutively, and cross the 24 time zones of the earth annually on the night of December 24^th^ [12]. It is unclear whether he relies on over the counter supplements (e.g., melatonin, which has recently been shown to be effective in shift workers [13]) or prescription pharmaceutical agents to manage the temporal transitions, nor whether he must resort to the use of stimulants (e.g., caffeine [14]) to manage such long working hours.

### Heat stress

Santa Claus, being acclimatized to the colder climate of extreme Northern latitudes, could also be expected to suffer from heat stress when delivering presents in warmer climates (e.g., Honolulu). Little is known about the efficacy of his fur-lined red suit in thermal regulation, and carrying his sack of presents would reasonably increase his physical workload, exacerbating heat stress further. As his preferred route of entry into the homes of his clients is via the chimney, there is the additional consideration of work in hot and confined spaces, which we believe has not been well-characterized in terms of risk. Indeed, no less esteemed an agency than the United States National Institute of Occupational Safety and Health has previously called attention to the issues Santa may encounter in this and other domains [15]. As it has been previously established that Santa performs the majority of his job duties in a once-annual, approximately 36-h stretch [12], it is extremely unlikely that an opportunity exists for heat acclimatization training beforehand.

### The older worker and succession planning

In addition to the adverse cardiovascular effects of shiftwork on Santa’s health, one important and sadly unmodifiable cardiovascular risk factor is relevant—namely age. Santa’s exact age is uncertain (possibly due to lack of proper documentation, bringing up the uncomfortable spectre of whether Santa, indeed, has the proper work permits…but we shall concern ourselves with medical and not administrative matters). As depicted in the advertisements of a certain soft drink manufacturer [16], he appears to have been an older man since at least the 1930s. It is unclear whether Santa has ever been offered a workplace health promotion program geared towards helping older workers remain productive and contributing members of the workforce, nor whether succession planning has ever been discussed with Santa.

### Mental health

Santa’s decision making capability is typically accepted as adequate by his clients and his mental health is not usually called into question. According to popular legend and song he is, at least when performing his duties in the urban environment (“Santa Claus is coming to town” [17]) gifted with near-omniscience (“He sees you when you’re sleeping / He knows when you’re awake / He knows if you’ve been bad or good…”). However, he still apparently keeps written records and checks them repeatedly (“He’s making a list, Checking it twice”). While double checking is to be encouraged for certain safety-sensitive work tasks, the practice does beg the question of whether Santa is acting according to standard operating procedures, or whether he may instead display a tendency towards compulsive checking. Recent evidence does link work stress with many common mental disorders, including obsessive compulsive disorder [18].

Additionally, Santa’s overall mental health may be adversely impacted by years of having to operate under government scrutiny. The North American Aerospace Defense Command has openly admitted that Santa’s December travels are tracked, in an annual tradition of government surveillance a-la “Big Brother” dating back to 1955 [19].

### Workshop ergonomics

Santa would be considered a giant among elves. While the average height of the latter has not been well established in the published literature, it is commonly accepted that they are diminutive in stature. Therefore, in a workplace where workstations and equipment are sized to comfortable dimensions for elvenkind, it is highly probable that Santa would find himself outside the 95^th^ percentile of elven height. As a consequence, he would likely require a dedicated ergonomic assessment and adjustment of the shared work environment in order to prevent progressive-onset musculoskeletal disorders.

## Conclusions

It has previously been reported that there is no standardized requirement for Santa to have a medical check-up, other than pre-employment drug screening [1]. Given the above-mentioned concerns from an occupational health perspective, we feel that it is time to adopt an evidence-based approach to develop, firstly, a comprehensive workplace occupational health program for Santa, and, secondly, a standardized and reproducible protocol for assessment of Santa’s fitness for work. However, we are sensitive to recent trends emphasizing workplace inclusiveness, and would not want to single out Santa for special treatment, thereby possibly contributing to workplace stigma. We therefore additionally suggest that future research directions include efforts to similarly explore and develop recommendations concerning the occupational health of reindeer and elves.
